# Leukocyte Esterase Strip Quantitative Detection Based on RGB Photometry is a Probable Method to Diagnose Periprosthetic Joint Infection: An Exploratory Study

**DOI:** 10.1111/os.13667

**Published:** 2023-02-13

**Authors:** Qing‐yuan Zheng, Peng Ren, Long Cheng, Hao Liu, Runkai Zhao, Yicun Lv, Zongjie Geng, Kuan Lu, Ming Ni, Guo‐qiang Zhang

**Affiliations:** ^1^ Medical School of Chinese PLA Beijing China; ^2^ Department of Orthopedics, the First Medical Center Chinese PLA General Hospital Beijing China; ^3^ Department of Orthopedics, the Fourth Medical Center Chinese PLA General Hospital Beijing China

**Keywords:** Chronic Periprosthetic Joint Infection, LE Strip Test, Quantitative Detection, RGB Photometry

## Abstract

**Objective:**

Leucocyte esterase (LE) strip test is the most rapid, convenient, and cheap method to diagnose chronic periprosthesis joint infection (PJI). However, the determination of LE strip mainly relies on colorimetric method with strong subjectivity, which leads to low diagnostic accuracy. Therefore, we try to convert LE strip images into digital data through the RGB photometric system to achieve objective diagnosis. This method will greatly improve the accuracy of LE strip detection and diagnosis of PJI.

**Methods:**

From January 2021 to September 2021, 46 patients with suspected PJI after total hip and knee arthroplasty underwent diagnostic joint puncture. After effective joint fluid samples were harvested, they were divided into original fluid and centrifuged fluid for LE strip detection. Real‐time images of LE strip were taken at 90 s, 3 min, 5 min, 10 min, and 15 min after sampling, and their brightness (*Y*) was obtained after they were input into an RGB photometric system. Grouping was based on centrifugation, infection, and time points, and then the differences in brightness among groups were compared. The correlation between LE strip image brightness and WBC count was evaluated. Student *t*‐test was used for the parametric data and chi‐square test for qualitative data. Simple linear regression was utilized to analyze the correlation between brightness and WBC count in each group.

**Results:**

Included were 19 cases of PJI and 27 Non‐PJI subjects diagnosed against ICM2018 diagnostic criteria. The brightness was lower in the PJI group than in Non‐PJI group (*p* < 0.05). The brightness of the uncentrifuged group was lower than that of the centrifuged group (*p* < 0.05). Irrespective of centrifugation or infection, the brightness of LE strip decreased with the exposure time after sampling. The brightness of LE strip was correlated with WBC count at different time points, with the correlation being strongest 5 min after sampling (*R* ^2^ (5 min) = 0.86, *p* < 0.0001). The correlation between LE strip brightness and WBC count was also found in the centrifugation group, with the correlation being most robust 15 min after sampling (*R* ^2^ (15 min) = 0.73, *p* < 0.0001).

**Conclusion:**

A remarkable correlation was found between LE strip brightness and the WBC count. It is feasible to directly quantify LE strip image on a RGB photometer to achieve quantitative detection of LE strip to diagnose PJI.

## Graphical abstract

As shown in the Graphical Abstract Image, our study confirmed that there was a good correlation between the WBC count and the brightness value of the leucocyte esterase strips at the time points of 90 s, 3 min, 5 min, 10 min, and 15 min, and the correlation was the best at the time point of 5 min. It may be possible to provide a reliable method for the quantitative detection of leucocyte esterase strips by using RGB photometric system and to establish the correlation between leucocyte esterase strips brightness and WBC count, thus improving the accuracy of rapid diagnosis of PJI.

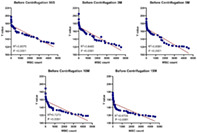

## Introduction

The newly issued International Consensus Meeting (ICM2018) criteria further highlights the importance of synovial fluid analysis in periprosthesis joint infection (PJI) diagnosis.^1^ Among the alternatives of synovial fluid analyses, LE strip detection is the most convenient, rapid, and accurate method, and works as effectively as leukocyte count and α‐defensin detection (ICM2018 score is 3 points).^2^ However, the colorimetric detection of LE strips still relies on the naked eye for interpretation, which is subjective and easily interfered by the external factors, and some strips might present borderline shades and cannot be effectively distinguished by the naked eye.^3^

At present, fully automatic urine analysis system is widely used in urinary tract infection detection, which substantially improves the detection efficiency.^4^ Jun Koh et al. confirmed that the results of the visual colorimetric observation were well‐consistent with those of automatic machine reading for the diagnosis of PJI.^5^ Nonetheless, this kind of equipment achieves semi‐quantitative measurement^6^ of the color change of LE strips by receiving dual‐wavelength light from a spherical integrator. In principle, the color change of LE strip results from the diazotization reaction between the leukocyte esterase secreted by leukocytes and the substrate on the strips pad. The neutral particles release esterase, which reacts with the substrate on the strips to produce indole phenol. Under the oxidation condition, two indophenol complexes form indigo that can serve as an indicator of leukocyte esterase activity.^7^ Leukocyte esterase activity is generally believed to be directly proportional to the leukocyte count. Therefore, there may exist a correlation between the color brightness (*Y* value) of the LE strip pad and leukocyte count, which may allow a shift from the current qualitative detection to quantitative detection of LE strips.^8^

The main purpose of the present study was to: (i) determine whether the brightness value of the leucocyte esterase strips image can effectively distinguish PJI from Non‐PJI; (ii) determine the influence of centrifugation, time, and other factors on the brightness value of the leucocyte esterase strips image; (iii) determine the correlation between the brightness value of the leucocyte esterase test paper image and the leukocyte count, so as to provide a theoretical basis for further quantitative detection.

## Materials and Methods

### Inclusion and Exclusion Criteria

The inclusion criteria were: (i) patients with suspicious PJI after hip and knee arthroplasty (i.e., having fever, swelling, unexplained pain, prosthesis loosening, and elevated erythrocyte sedimentation rate, abnormal increase of CRP, etc.); (ii) patients who were scheduled to undergo revision hip and knee surgery; (iii) the patient agreed to undergo arthrocentesis without contraindications; (iv) the patient had received antibiotics within 2 weeks prior to the arthrocentesis; (v) this study is a prospective exploratory study.

The exclusion criteria were: (i) joint cavity puncture failed; (ii) white blood cell count could not be collected due to the interference of viscous joint fluid or red blood cells; (iii) against ICM018 diagnostic criteria, the patients scored 3–5 or the diagnosis could not be confirmed due to lack of information.

### Demographics

Our institution's research ethics board approved this prospective exploratory study (Ethics No. S2022‐009‐02). We enrolled patients who were suspected of PJI after hip or knee arthroplasty from May 2020 to September 2020 at a single institution. Ultimately, 46 patients were included. According to ICM2018 diagnostic criteria, 19 of the patients were categorized as infected (score ≥6) and 27 were classified as uninfected (score ≤2).^1^ The clinical records of the patient's information that included age, body mass index (BMI), gender, surgery type (knee or hip), comorbidity, and conventional diagnostic indicators of PJI were showed in Table 1.

**TABLE 1:** Cohort characteristics

Characteristic	Cohort		*p*‐value
	PJI (*N* = 19)	non‐PJI (*N* = 27)	
Sex^a^			0.22
Male	9 (47.4%)	8 (29.6%)	
Female	10 (52.6%)	19 (70.4%)	
Age^b^ (years)	64.58 ± 8.09	67.78 ± 7.75	0.192
BMI^b^ (kg/m^2^)	25.49 ± 6.05	25.74 ± 2.65	0.819
Joint^a^			0.877
Hip	3 (15.8%)	5 (18.5%)	
Knee	16 (84.2%)	22 (81.5%)	
Immune system disease^a^ ^,^ ^c^			0.877
With	3 (15.8%)	5 (18.5%)	
Without	16 (84.2%)	22 (81.5%)	
Serum CRP^b^ (mg/L)	2.53 ± 1.33	0.37 ± 0.22	<0.001
Serum ESR^b^ (mm/h)	48.26 ± 32.62	14.26 ± 7.71	<0.001
WBC count^b^ (10^9^/μl)	1893.79 ± 1576.39	659.56 ± 834.73	<0.001
Bacterial culture^a^			<0.001
Positive	12 (63.2%)	2 (7.4%)	
Negative	7 (36.8%)	25 (92.6%)	
ICM2018 Score^b^	9.00 ± 2.97	0.59 ± 0.91	<0.001

Abbreviations: BMI, body mass index; CRP, C‐reactive protein; ESR, erythrocyte sedimentation rate; WBC count, white blood cell count.

^a^ The values are given as the number of cases.

^b^ The values are given as the mean, with the range in parentheses.

^c^ “Immune System Disease” includes a history of rheumatoid arthritis (four cases), a history of ankylosing spondylitis (two cases), a history of sicca syndrome (one case), and systemic lupus erythematosus (one case).

### Testing Process

#### Joint Fluid Acquisition and Treatment

All the diagnostic arthrocentesis were performed in the outpatient operating room of our hospital, and all the operation steps followed the guidelines of the standardized arthrocentesis and were tailored to specific anatomical locations. The collected synovial fluid was divided into two equal portions, with one portion being transferred to a common centrifuge tube by means of a syringe and centrifuged as recommended by Aggarwal et al. (SCILOGEX D3024, at 5140g and for 180 s).^9^

#### LE Strips Image Data Acquisition

Two operators, separately, took 1000 μl synovial fluid from the stock solution and centrifugal solution, using a pipette gun, and applied it to LE test pads (10 Pa; ARKRAY). When two operators applied the samples for the first time, one assistant immediately used a stopwatch to record the time elapsed, and signaled the operator to continue applying samples for another four times 10 min, 5 min, 3 min, and 1.5 min (90 s) after. At the end of the 15‐min countdown, the operator immediately used the high‐definition camera fixed above the test strip to take the picture of the test strip (the camera was 15 cm over the test strip. Each picture was taken in a fixed position, and the focal length and visual field were calibrated to ensure that the external conditions were consistent).

The LE strip test for diagnosing urinary tract infection required 90 s to obtain a result. In accordance with the manufacturer's directions, the LE strip test with a 3‐min reading time has been used for the diagnosis of PJI. Previous studies have shown that the LE strips be read 5 min after application and before centrifugation, while 10 min after application may be the appropriate time for reading the results of LE strip tests post‐centrifugation. In addition, the result of LE strip did not change after 15 min.^10^ Therefore, we select the above five time points to acquire image data.

After obtaining the image of LE strips, the leukocyte esterase strips were cut into a square of 80 × 80 pixels (with clear boundary) and grouped into different image data sets in terms of centrifugation/stock solution and sampling time points (1.5, 3, 5, 10, and 15 min). Then, the image data sets were inputted into an RGB photometric system, RGB values were obtained, and brightness (*Y* value) was automatically calculated according to the following formula:

Y=0.299×R+0.587×G+0.114×B. When Y value decreased, the brightness of strips decreased (i.e., the darker the LE strips, the stronger the activity of leukocyte esterase).

#### Leukocyte Counting

Leukocyte counts of 46 patients were obtained from the clinical laboratory of our hospital.

Sample collection for leukocyte counting: After the joint fluid was harvested, an appropriate amount of joint fluid (>1 ml) was immediately taken and transferred into a transparent sterile reagent tube, with the nozzle sealed. Afterwards, the samples were sent to the clinical laboratory center of our hospital.

Leukocyte counting equipment and reagents: Sysmex XN (Sysmex) automatic modular hematology and humoral analyzer and the reagents were used.^11^

Leukocyte counting method: The laboratory technicians operated by following the instructions of the analyzer, and the Body Fluid module was selected for counting the white blood cells in the joint fluid samples. After complete shaking, the specimens were tested, and the final results were recorded.

#### Microbial Cultivation

Part of the obtained joint fluid (>1.0 ml) was respectively injected into aerobic/fungal culture flasks (Bact/Alertpfpediatricfan) and anaerobic culture flasks (Bact/Alert Fafan) for bacterial culture. The microbiology department of our hospital issued relevant results. BacT/Alert 3D (BioMérieux) blood culture system was used for the microbial culture of joint fluid, and the culture lasted for 2 weeks (14 days) or till appearance of positive result of microorganism, whichever came first. VITEK‐MS (BioMérieux) system was used for microbial identification.

### Statistical Analysis

Categorical variables were expressed as frequencies and percentages, and continuous variables were expressed as mean ± standard deviation. Student *t*‐test was used to evaluate the parametric data, while chi‐square test (corrected chi‐square test) was employed to analyze the non‐parametric variables for inter‐group comparison (IBM SPSS Statistics, version 23.0.0.0). Simple linear regression was utilized to analyze the correlation between brightness (*Y* value) and leukocyte count in each group. The simple linear regression curve was plotted by using GraphPad Prism package (version 8.3; GraphPad Software). The statistical significance was defined as *p* < 0.05.

## Results

### General Results

A total of 46 patients were divided into a PJI group (19 cases) and a non‐PJI group (27 cases), and demographic information is shown in Table 1.There were no statistically significant differences in male/female ratio, age, body mass index (BMI), immune system diseases, and the ratio of hip and knee joints between the two groups. There were significant differences in serum CRP, ESR, microbial culture results, leukocyte count, and ICM2018 score between the two groups (*p* < 0.01).

### Bacterial Culture

In PJI group, the bacterial culture of 12 cases (12/19, 63.16%) yielded positive results and two cases were positive for *Candida corneae/Candida parapsilosis; Staphylococcus/Klebsiella pneumoniae*. Two cases (2/27, 7.41%) in non‐PJI group were positive for *Micrococcus luteus* and *Staphylococcus epidermidis*, which were two common contaminating bacteria. There were statistically significant differences in the results of bacterial culture between the two groups (*p* < 0.01). (Table 2).

**TABLE 2:** Details of detected microorganisms

Detected microorganisms	Cohort	
	PJI (*N* = 12)	non‐PJI (*N* = 2)
*Candida corneae*	1^a^	
*Candida parapsilosis*	1^a^	
*Staphylococcus epidermidis*	4	1
*Staphylococcus aureus*	1	
*Enterococcus faecalis*	2	
*Staphylococcus lugdunensis*	1	
*Klebsiella pneumoniae*	1^a^	
*Staphylococcus hominis*	2^a^	
Gram‐positive Bacilli	1	
*Micrococcus luteus*		1

^a^ Two kinds of bacteria were cultured simultaneously in two samples, one case was Candida Cornea with *Candida parapsilosis*, and the other was *Klebsiella Pneumoniae* with Staphylococcus Hominis.

### RGB Luminance Data

#### Infected Group (PJI) Vs. Non‐infected Group (Non‐PJI)

**FIGURE 1:**
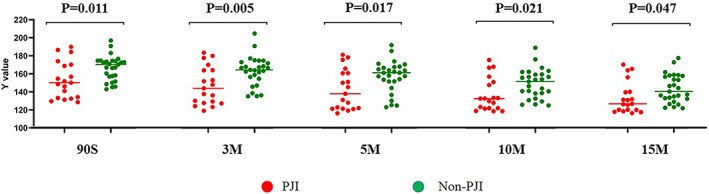
Comparison of LE strip brightness between PJI groups at different time points before centrifugation

Ninety seconds after the application of the synovial fluid, the brightness (*Y* value) of PJI group was 153.24 ± 20.34 and the *Y* value of non‐PJI group was 166.49 ± 13.67 (*p* < 0.05). When the synovial fluid was spotted for 3 min, there was a statistically significant difference in the *Y* value between PJI group (146.8 ± 21.02) and non‐PJI group (162.99 ± 16.36) (*p* < 0.05). When the synovial fluid was spotted for 5 min, the *Y* value of PJI group was 142.56 ± 22.30 and that of non‐PJI group was 157.01 ± 17.31, and the difference was statistically significant (*p* < 0.05). Ten minutes after spotting of the synovial fluid, the *Y* value of PJI group was 136.76 ± 18.42 and that of non‐PJI group was 149.03 ± 16.04, and the difference was statistically different (*p* < 0.05). Fifteen minutes after spotting the synovial fluid, the *Y* value of PJI group was 133.45 ± 17.71 and that of non‐PJI group was 143.54 ± 15.63, and the difference was statistically significant (*p* < 0.05). (Figure 1).

**FIGURE 2:**
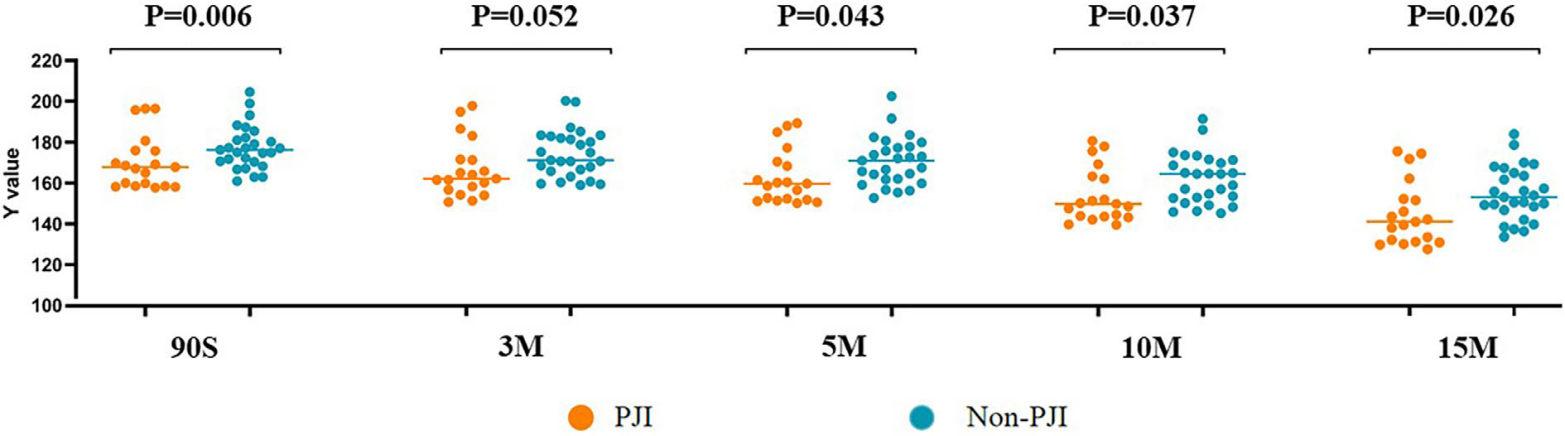
Comparison of LE strip brightness between PJI groups at different time points after centrifugation

Ninety seconds after the application of articular centrifugal fluid, no statistically significant difference in *Y* value was found between PJI group (170.48 ± 13.27) and the non‐PJI group (177.30 ± 10.64) (*p* = 0.060). When the synovial fluid sample was centrifuged for 3 min, no statistically significant difference in the *Y* value was revealed between PJI group (166.76 ± 14.12) and the non‐PJI group (174.31 ± 11.52) (*p* = 0.052). When the synovial fluid sample was centrifuged for 5 min, the *Y* value of PJI group was 162.90 ± 13.19 and that of the non‐PJI group was 170.61 ± 11.74, and the difference was statistically significant (*p* < 0.05). When the synovial fluid sample was centrifuged for 10 min, the *Y* value of PJI group was 153.89 ± 13.40 and that of the non‐PJI group was 162.04 ± 12.17, and the difference was statistically significant (*p* < 0.05). When the synovial fluid sample was centrifuged for 15 min, the *Y* value of PJI group was 144.77 ± 15.81 and that of the non‐PJI group was 154.59 ± 13.06, with the difference being statistically significant (*p* < 0.05). (Figure 2).

#### Before Centrifugation Group (BC) Vs. after Centrifugation Group (AC)

**FIGURE 3:**
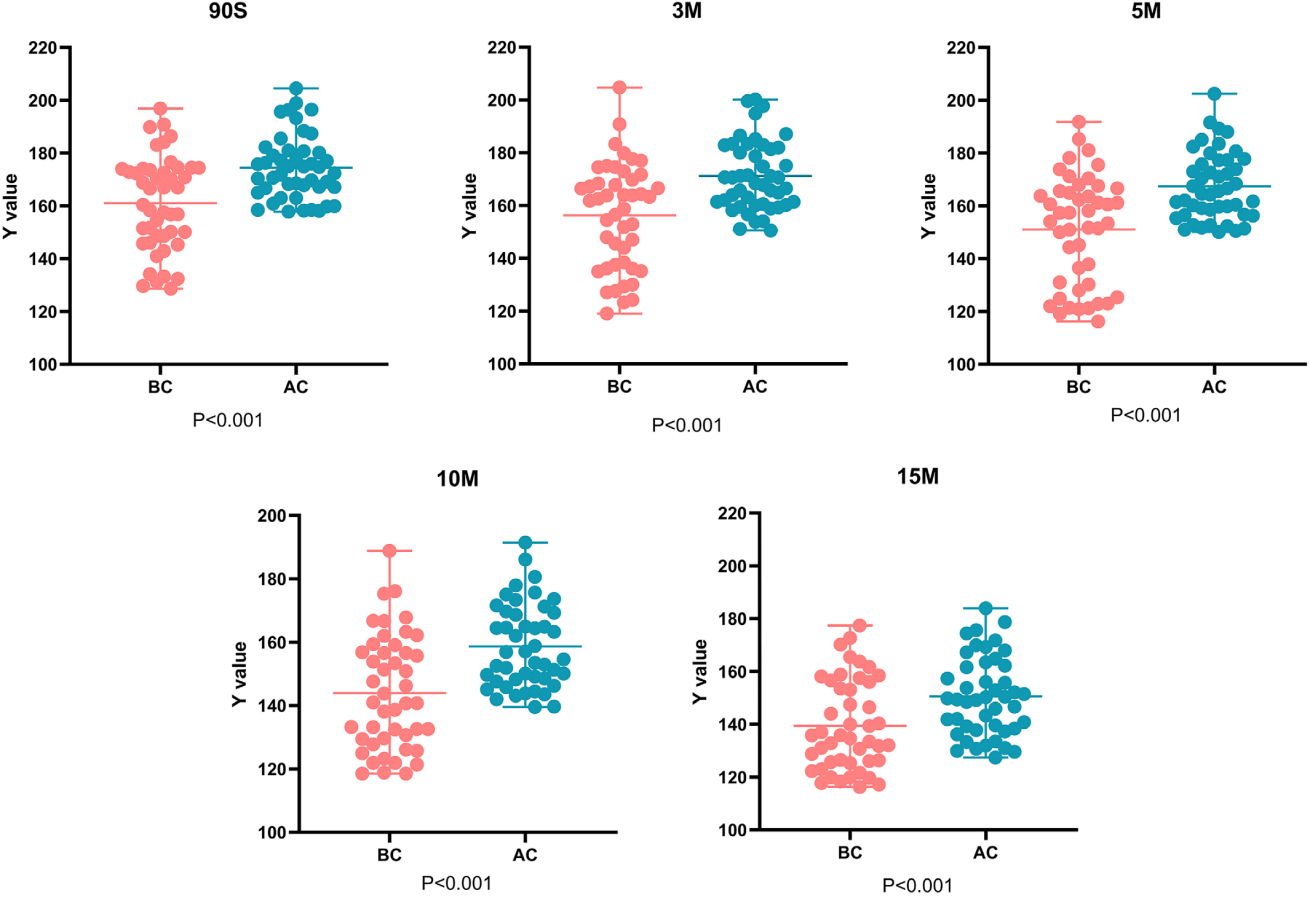
Comparison of LE strip brightness at different time points before and after centrifugation

Synovial fluid sample before and after centrifugation (BC and AC groups) were sampled on LE strips pad. When the synovial fluid sample was spotted for 90 s, the brightness (*Y* value) of BC group was 161.01 ± 17.80 and the *Y* value of AC group was 174.48 ± 12.14 (*p* < 0.001). When the synovial fluid sample was spotted for 3 min, the *Y* value of BC group was 156.30 ± 19.91 and the *Y* value of AC group was 171.19 ± 13.06, and the difference was statistically significant (*p* < 0.001). When the synovial fluid sample was spotted for 5 min, the *Y* value of BC group was 151.04 ± 20.58 and that of AC group was 167.43 ± 12.80 (*p* < 0.001). Ten minutes after the synovial fluid sample spotting, a statistically significant difference was found in the *Y* value between BC group (143.96 ± 17.94) and AC group (158.67 ± 13.18) (*p* < 0.001). When the synovial fluid sample was spotted for 15 min, the *Y* value of BC group was 139.37 ± 17.09 and that of AC group was 150.53 ± 14.91, with the difference being statistically significant (*p* < 0.001). (Figure 3).

### Comparison of Different Times Groups

The samples were dropped into synovial fluid before and after centrifugation on the LE strips pad and were divided into different groups in terms of the time after sampling (i.e., 90 s, 3 min, 5 min, 10 min, and 15 min groups).

Statistically significant differences in the brightness *Y* of the LE strips were found among BC groups of different time lengths (90 s, 3 min, 5 min, 10 min, and 15 min after spotting) (*p* < 0.001), and the brightness of the LE strips in BC group gradually decreased with the exposure time. There existed statistically significant differences in *Y* values of the brightness of the LE strips in the AC groups of different time lengths (90 s, 3 min, 5 min, 10 min and 15 min after spotting) (*p* < 0.001), and the brightness of the LE strips in AC groups gradually dropped with the exposure time (Table 3).

**TABLE 3:** The effect of time on the intensity of LE Strip

Classification	Times								
	90 S		3 M		5 M		10 M		15 M
Before centrifugation	161.01 ± 27.60		156.30 ± 27.19		151.04 ± 26.88		143.96 ± 24.29		139.37 ± 21.06
Statistic value		*t* = 8.149		*t* = 10.579		*t* = 8.745		*t* = −11.084	
*p*‐value		<0.001		<0.001		<0.001		<0.001	
After centrifugation	174.48 ± 8.78		171.19 ± 11.08		167.43 ± 9.17		158.67 ± 11.00		150.53 ± 13.16
Statistic value		*t* = 7.772		*t* = −11.141		*t* = 46.807		*t* = −21.172	
*p*‐value		<0.001		<0.001		<0.001		<0.001	

Abbreviations: S: second; M: minute.

### *Correlation Result between Brightness (*Y*) of LE Strips and WBC Count*

Centrifuge anterior articular fluid (Before Centrifugation; BC) Correlation between brightness (*Y*) of Le strips and WBC Count.

**FIGURE 4:**
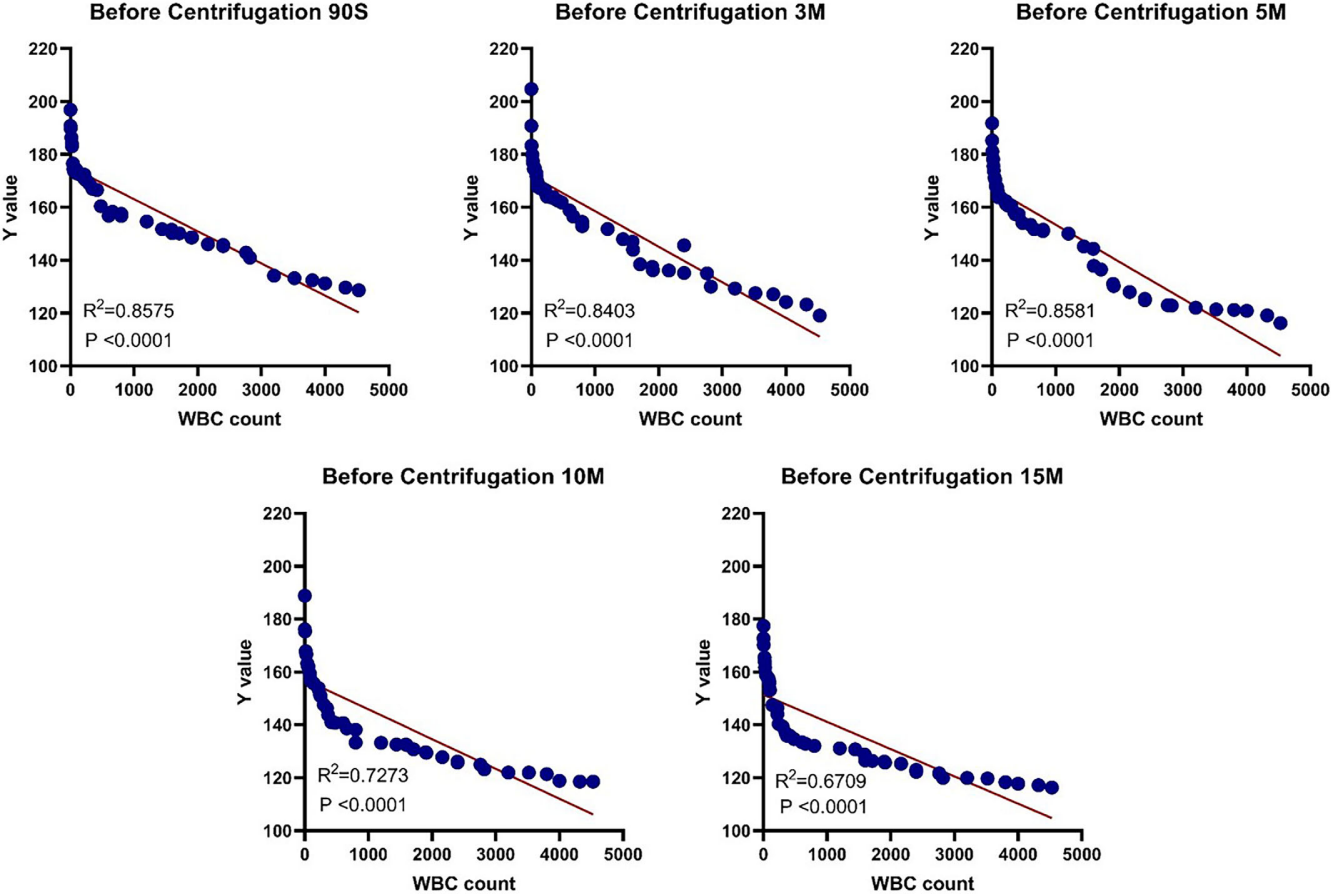
Correlation between WBC count and LE strip brightness at different time points before centrifugation

There existed a correlation between the brightness (the *Y* value) of the LE strips before the joint centrifugation and the white blood cell counts 90 s, 3 min, 5 min, 10 min, and 15 min after dispensing (correlation coefficient: *R* ^2^ (90 s) = 0.8575, *p* < 0.0001; *R* ^2^ (3 min) = 0.8403, *p* < 0.0001; *R* ^2^ (5 min) = 0.8581, *p* < 0.0001; *R* ^2^ (10 min) = 0.7273, *p* < 0.0001; *R* ^2^ (15 min) = 0.6709, *p* < 0.0001). The correlation was strongest 5 min after sampling among the five time lengths (*R* ^2^ (5 min) = 0.8581, *p* < 0.0001) (Figure 4).

Centrifuge the posterior articular fluid (After Centrifugation; Ac) correlation between brightness (*y*) of le strips and WBC Count.

**FIGURE 5:**
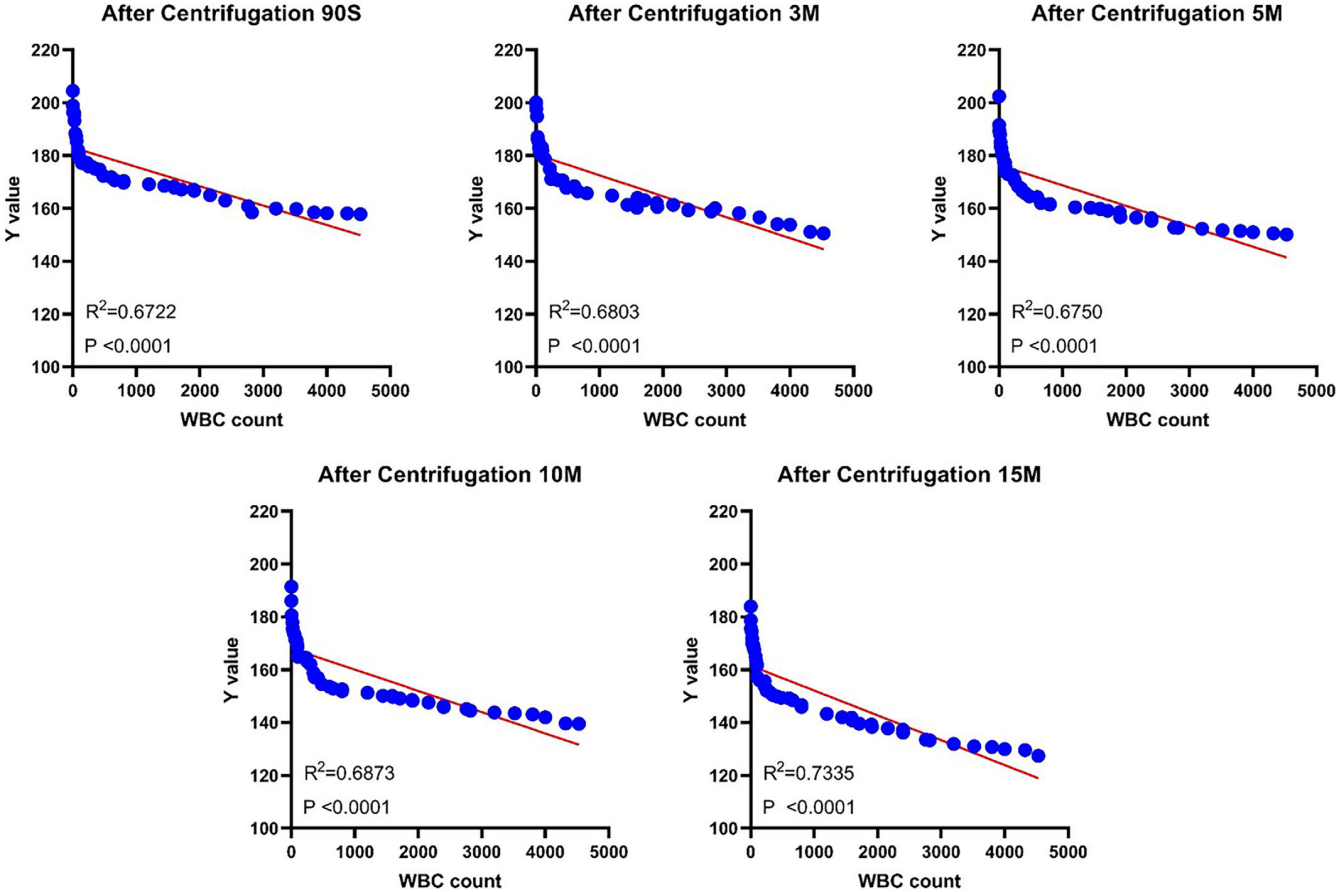
Correlation between WBC count and LE strip brightness at different time points after centrifugation

A correlation was found between the brightness (*y*) of LE strips and the white blood cell count 90 s, 3 min, 5 min, 10 min and 15 min after the joint fluid centrifugation (correlation coefficient: *R* ^2^ (90 s) = 0.6722, *p* < 0.0001; *R* ^2^ (3 min) = 0.6803, *p* < 0.0001; *R* ^2^ (5 min) = 0.6750, *p* < 0.0001; *R* ^2^ (10 min) = 0.6873, *p* < 0.0001; *R* ^2^ (15 min) = 0.7335, *p* < 0.0001). The correlation 15 min after sampling was strongest among the five time lengths (*R* ^2^ (15 min) = 0.7335, *p* < 0.0001) (Figure 5).

## Discussion

In this study, we have found that digital data of leucocyte esterase strip images through the RGB photometric system can effectively differentiate between chronic PJI and non‐PJI. The variation trend of its quantitative value was also consistent with the trend that centrifugation and time affected the results of LE strips test. In addition, there was a correlation between the WBC count and the quantitative data of the leucocyte esterase strip image.

### Exploration of Quantitative Detection of LE Strips

In the field of joint fluid analysis for PJI diagnosis, Javad Parvizi and others took the lead by introducing LE strips with good results achieved.^12^ Wetterng et al. further confirmed the value of LE strips in PJI diagnosis.^13^ The LE strips are characterized by simplicity, portability, and rapidity. However, the value of the methods that rely on qualitative judgment and visual colorimetry undoubtedly was limited by the relatively low detection accuracy of LE strips.^3^ Quantitative evaluation with the LE strip results can effectively make up for this limitation. To attain the quantitative determination of LE strip results, some meaningful attempts have been made, especially in the fields of laboratory medicine and urology. First, automatic analysis system was developed and was successfully employed for the clinical diagnosis of urinary tract infection. This development has laid a solid groundwork for the quantitative analysis of LE strips.^6^, ^14^, ^15^, ^16^ Choi K et al. worked out a smart‐phone‐based LE strip detection technique and used it for emergency situations. The diagnostic accuracy rate of the LE strip detection reached 82.5%, which paved the way for the development of easier and more accurate of LE strip test.^17^ In the research of synovial fluid analysis, Jun Koh and others also did some trailblazing work for the application of automatic machine reading.^5^

In addition, earlier studies demonstrated that the reflectance data of leukocyte esterase concentrations could be verified with the WBC counts obtained by flow cytometry and vice versa.^18^, ^19^ In recent years, complementary metal oxide semiconductor (CMOS) technology was introduced into various strip tests.^20^, ^21^, ^22^ Oyaert et al. conducted a series of studies on automatic dry chemical analysis (on a Sysmex UC‐3500 urinary test strip reader) in combination with CMOS technology. Their results showed that the results of LE strips were quantifiable and could be mutually verified with leukocyte counts, which offered a feasible way to achieve quantitative detection of LE strips.^21^ However, the application of CMOS in these methods was still premised on the measurement of the light reflected from the surface of the strip pad, which was indirectly indicative of the intensity of the reaction color of the strips pad. With these methods, difference in light reflectance depends on the differences in substrate concentrations, and colorimetric assessment was accomplished by comparing the samples and the blank pad of the strips. Yang et al. combined smart phones with biosensors and developed a quantitative detection algorithm of strips based on human vision. The CIEDE2000 equation in CIELab color space model was employed to evaluate the difference in color change to realize the quantitative analysis of urine.^23^ However, these methods were of indirect nature in terms of color quantification and were subject to a great many factors.

On the basis of the previous research, we attempted to directly quantify the color change of LE strip. This quantified color change data is collected and analyzed in real time.

RGB photometric determination is an internationally accepted color quantification system. Brightness (*Y*) is derived from RGB values, which can accurately reflect the brightness^24^ of an object. With the LE strip test, the lower the brightness (*y*), the darker the image of the LE strips (darker purple). Furthermore, a higher activity of leucocyte esterase is indicative of a higher content of leucocytes. In this study, the samples were first divided into groups in terms of infection (PJI vs. non‐PJI), centrifugation (before and after centrifugation of the stock solution) and time points or lengths (90 s, 3 min, 5 min, 10 min, and 15 min after the sampling), and then the image brightness (*Y*) of LE strips was compared among the different groups.

### Chronic PJI Vs. Non‐PJI

First of all, before centrifugation, the brightness (*Y*) of LE strips of the synovial fluid (stock solution) in the infected (PJI) and non‐infected (non‐PJI) was significantly different at five different time points (90 s, 3 min, 5 min, 10 min, and 15 min after sampling) (*p* < 0.05). The brightness of synovial fluid LE strips was lower in the PJI group than in the non‐PJI group. This was consistent with the results of many other studies examining the role of WBC count in PJI diagnosis.^25^, ^26^ Nonetheless, in the centrifuged synovial fluid (centrifugal fluid), no statistically significant differences were found in the brightness (*Y*) between PJI group and non‐PJI group and among post‐spotting 90 s and 3 min groups (*p* = 0.060; *p* = 0.052). Our result showed that up to the 5 min after the sample was printed, the difference in the brightness (*Y*) of LE strips between the two groups was statistically significant (*p* < 0.05). The above results indicate that the centrifuged synovial fluid may take a longer reaction time to yield a better detection result.

### Effect of Centrifugation

Then, as to the influence of centrifugation, we found that at five different time points (90 s, 3 min, 5 min, 10 min, and 15 min after sampling), the brightness (*Y*) of the synovial fluid LE strips after centrifugation was significantly higher than that before centrifugation, and these differences were statistically significant (*p* < 0.001). This was in line with the conclusion reached by Rui Li et al. that centrifugation would lead to degradation of the reading of LE strips of synovial fluid.^27^

### Effect of Time

Furthermore, time also exerted an influence on the result of LE strips test results. With qualitative detection, the reading results of LE strips improved with the extension of exposure time. This phenomenon was further substantiated by the quantitative detection of LE strips based on RGB photometric measurement: we observed the results at five time points, i.e., 90 s, 3 min, 5 min, 10 min, and 15 min, and compared the brightness (*Y*) of the joint fluid LE strips before and after centrifugation (of the stock solution or the centrifuge solution) at these five time points. The brightness (*y*) was lower in the longer time group than in the shorter time groups of the previous group, and the differences were statistically significant between different time points (*p* < 0.001 for all). This further proved that the color of LE strips does exist and becomes increasingly darker over exposure time.

### Study of Correlation

Additionally, this study also confirmed that the brightness (*Y*) of LE strips before and after the centrifugation of synovial fluid was correlated with WBC count at five different time points (90 s, 3 min, 5 min, 10 min, and 15 min after sampling).

Five minutes after sampling, the correlation (*R* ^2^ = 0.8581) between the image brightness of LE strips in synovial fluid before centrifugation was stronger than that at other time points. However, 10 and 15 min after spotting, the correlation between image brightness of LE strips of synovial fluid before centrifugation and white blood cell count (*R* ^2^ = 0.7273; *R* ^2^ = 0.6709) became significantly weaker, which might be ascribed to longer detection interval, which prolonged the exposure time of LE strips to the air. One possibility is that there is peroxide^28^ in the diazonium salt reaction process, and the other possibility is the volatilization of phenols^4^ in the reaction. Under the combined effect of these two factors, the result of the qualitative LE strip tests can be maintained at a certain level, but with quantitative detection, deviation may result, leading to a weaker correlation with leukocyte count.

Unlike the synovial fluid before centrifugation, the brightness of LE strip image of the synovial fluid after centrifugation was found to bear a relation with the WBC count (*R* ^2^ = 0.7335), with the association being the strongest 15 min after sampling.

On the whole, the curve of correlation between image brightness (*Y*) and leukocyte count in synovial fluid LE strips before and after centrifugation is clearly distinguished at the node of 1000 × 10^9^/μl. Leukocyte count was obviously of non‐linear nature within 1000 × 10^9^/μl, while the leukocyte count exceeds 1000 × 10^9^. Since the WBC count in the range of 0–1000 × 10^9^/μl is of little significance for PJI diagnosis, the linear regression coefficient can, to a certain extent, reflect the correlation between the brightness of LE strips and leukocyte count, so we did not analyze it in sections.

### Strengths and Limitations

Our study provides a feasible method for the quantitative detection of chronic PJI based on LE strip test. This method not only retains the advantages of being simple, rapid, and cheap but also improves the objectivity and accuracy of LE strip test in the diagnosis of chronic PJI.

However, because the curve of correlation between them was partially non‐linear, as a result, we cannot use the correlation function between the brightness of the strips and the WBC count to directly accomplish the quantitative determination of LE strips. We believe the non‐linear nature of the curve might be attributed to the following facts: (1) the external environment (including external light, camera equipment settings, etc.) interferes with the collected images of LE strips. Although we made effort to fix the location and position to minimize the influence of the environment, a completely constant external environment could not be achieved; (2) the blood and other confounding factors interfered with the results of image brightness, though we ruled out samples that could not obtain accurate WBC cell counts due to the interference of red blood cells, under the background of direct analysis of image data and quantitative determination, even a small amount of mixed factors such as red blood cells might have an impact on the final result, which was almost inevitable.

Moreover, the correlation coefficient between image brightness (*Y*) and WBC count of LE strips of synovial fluid after centrifugation was lower than that before centrifugation, and centrifugation may affect the results of PJI diagnosis by LE strips.^26^ However, the centrifugation of synovial fluid is intended to remove blood mixing and other factors in synovial fluid, which seems to be unavoidable.^9^ In addition, in our experiment, the image data collection was too cumbersome, the grasp of time nodes still depends on manual operation, and the equipment used (such as external cameras, etc.) does not meet the clinical requirement in terms of convenience.

### Conclusion

To sum up, we believe that it is feasible to attain quantitative detection of LE strips on the basis of direct analysis of LE strips, and the good correlation between the image brightness (*Y*) of LE strips and WBC count provides a theoretical basis for the realization of this goal.

#### Author's Contribution

Q.Y.Z., L.C., and P.R. conducted the survey and made the evaluation. G.Q.Z. gave the inspiration for the survey. Q.Y.Z., L.C., and P.R. wrote the article. All authors have read and approved the final submitted manuscript.

## Funding Information

This work was supported by the Beijing Science and Technology Project (Z211100002921046).
